# Statistical method for mapping QTLs for complex traits based on two backcross populations

**DOI:** 10.1007/s11434-012-5279-8

**Published:** 2012-07

**Authors:** ZHU ZhiHong, HAYART Yousaf, YANG Jian, CAO LiYong, LOU XiangYang, XU HaiMing

**Affiliations:** 1 Agronomy Department, College of Agriculture and Biotechnology, Zhejiang University, Hangzhou 310058, China; 2 Department of Mathematics, Statistics and Computer Science, NWFP Agricultural University Peshawar, Peshawar 25130, Pakistan; 3 China National Rice Research Institute, National Center for Rice Improvement, State Key Laboratory of Rice Biology, Hangzhou 310006, China; 4 Department of Biostatistics, School of Public Health, University of Alabama at Birmingham, Birmingham, Alabama 35294, USA

**Keywords:** QTL mapping, double backcross populations, mixed linear model, epistasis, QTL-by-environment interaction

## Abstract

Most important agronomic and quality traits of crops are quantitative in nature. The genetic variations in such traits are usually controlled by sets of genes called quantitative trait loci (QTLs), and the interactions between QTLs and the environment. It is crucial to understand the genetic architecture of complex traits to design efficient strategies for plant breeding. In the present study, a new experimental design and the corresponding statistical method are presented for QTL mapping. The proposed mapping population is composed of double backcross populations derived from backcrossing both homozygous parents to DH (double haploid) or RI (recombinant inbreeding) lines separately. Such an immortal mapping population allows for across-environment replications, and can be used to estimate dominance effects, epistatic effects, and QTL-environment interactions, remedying the drawbacks of a single backcross population. In this method, the mixed linear model approach is used to estimate the positions of QTLs and their various effects including the QTL additive, dominance, and epistatic effects, and QTL-environment interaction effects (QE). Monte Carlo simulations were conducted to investigate the performance of the proposed method and to assess the accuracy and efficiency of its estimations. The results showed that the proposed method could estimate the positions and the genetic effects of QTLs with high efficiency.

In crop breeding, most target traits are quantitative traits, also known as complex traits. It is crucial to determine the genetic architecture of complex traits to understand their biological mechanisms and to plan efficient genetic improvement strategies. To map quantitative trait loci (QTLs), appropriate data are required, including molecular markers and phenotypic traits from a well-designed experiment. The commonly used mapping populations in plants include F2, backcross (BC), double haploid (DH), and recombinant inbred (RI) populations. Of these, the F2 population provides the most abundant genetic information [1]. However, the F2 population is temporary, the individuals differ from each other in their genetic constitution, and they cannot be duplicated unless somatic cell cloning is applied. Thus, it is not possible in a QTL study to conduct multiple environmental experiments with an F2 population to analyze QTL × environment (QE) interactions. Furthermore, all individuals in different environments need to be genotyped, which is expensive in terms of time, labor, and money. The BC population shares the same features as the F2 population mentioned above; as a result of less segregation information for genotypes, additive effects cannot be distinguished from dominance effects, and some types of epistatic effects are also confounded [2]. To investigate QTL effects across different environments, immortal DH and RI populations have been developed and widely applied in QTL mapping for many species [3–7]. Since DH and RI populations are homozygous, their marker data can be used repeatedly with phenotypes of quantitative traits observed in different locations and years under various experimental designs. However, these populations cannot be used to analyze dominance effects and some types of dominance-related epistatic effects, which play important roles in hybrid heterosis. In the present study, we tested an experimental design including a permanent double backcross population derived from crossing DH or RI lines to both the homozygous parents. The advantage of such populations is that identical mapping populations can be replicated as necessary.

There remains a great challenge in methodology for analyzing epistasis and QE interactions, two principal genetic components of QTL effects. The importance of epistasis affecting complex quantitative traits has been well documented in numerous classical quantitative genetic studies [8,9]. Several QTL studies have also indicated that epistasis is an important genetic basis for complex traits such as grain yield and its components, as well as heterosis and inbreeding depression [10–13]. The QE interaction is another important component for quantitative traits [14–20], but none of the previous methods has integrated these two parts into one unified mapping framework. Wang et al. [2] proposed a method based on a mixed linear model approach to tackle this problem for a DH or RI population; this method was later extended to a permanent F2 population [21]. However, those methods have some disadvantages in cofactor selection for controlling background genetic effects, false positive rates, and computational tractability. Therefore, a full-QTL model was proposed for systematically mapping QTLs underlying complex traits. This model was sufficiently flexible to include the effects of multiple QTLs, epistasis, and QE interactions [22].

Here, we propose a method under the framework of a mixed linear model based composite interval mapping (MCIM) for immortal double backcross populations. The proposed method uses the approach of the full QTL mapping model described by Yang et al. [22]. Monte Carlo simulations were carried out to assess the performance of the method.

## 1 Methods

### 1.1 Genetic model

The proposed genetic model is based on the mixed linear model. The mapping population consists of double back-cross populations derived from crossing DH or RI lines with both of the homozygous parents. The mapping population is used to map *s* segregating QTLs simultaneously with additive, dominance, and additive × additive epistatic effects, as well as their interaction effects with the environment, in which *t* pairs of QTLs are involved in epistatic interactions. The mixed linear model for the phenotypic value of the *k*th individual in the *h*th environment (*y_hk_*) can be expressed as follows (*h* = 1, 2, …, *p; k* = 1, 2, …, *n_h_*)

(1) $$y_{hk}=\mu \sum_{i}^{s}a_{i}x_{A_{ki}}+\sum_{i}^{s}d_{i}x_{D_{ki}}+\sum_{i,j\in (1,2,\cdots ,s);i\neq j}^{t}{aa}_{ij}x_{{AA}_{\mathrm{kij}}}+e_{h}+\sum_{i}^{s}{ae}_{hi}x_{A_{ki}}+\sum_{i}^{s}{de}_{hi}x_{D_{ki}}+\sum_{i,j\in (1,2,\cdots ,s);i\neq j}^{t}{\mathrm{aae}}_{\mathrm{hij}}x_{{AA}_{\mathrm{kij}}}+\varepsilon_{hk},$$

where *μ* is the population mean; *a_i_* and *d_i_* are the fixed additive and dominance effects of *Q_i_* (*i*th QTL), respectively; *aa_ij_* is the additive × additive epistatic effect (fixed) between *Q_i_* and *Q_j_*. *x*_*A*_*ki*__, *x*_*D*_*ki*__, and *x*_*AA*_*kij*__ are the coefficients of the above QTL effects that depend on the observed genotypes at the marker loci (let *M_i_*_−_ and *M_i+_* be two markers flanking *Q_i_* and *M_j_*_−_, *M_j+_* be markers flanking *Q_j_*, respectively) and the recombination frequencies (denoted by *r*_*M*_i−_*Q*_i__, *r*_*Q*_*i*_*M*_i+__ and *r*_*M*_j−_*Q*_j__, *r*_*Q*_j_*M*_j+__); *e_h_* is the random effect of the *h*th environment, $e_{h}\sim (0,\sigma_{E}^{2})$; *ae_hi_* is the random additive × environment interaction effect with coefficient *x*_*A*_*ki*__, ${ae}_{hi}\sim (0,\sigma_{A_{i}E}^{2})$; *de_hi_* is the random dominance × environment interaction effect with coefficient *x*_*D*_*ki*__, ${de}_{hi}\sim (0,\sigma_{D_{i}E}^{2})$; *aae_hij_* is the random additive-additive epistasis × environment interaction effect with coefficient *x*_*AA*_*kij*__ (=*x*_*A*_*ki*__ *x*_*A*_*kj*__), ${\mathrm{aae}}_{\mathrm{hij}}\sim (0,\sigma_{{AA}_{ij}E}^{2})$; *ε_hk_* is the random residual effect, $\varepsilon_{hk}\sim (0,\sigma_{\varepsilon }^{2})$.

Model (1) can be expressed in the following matrix form for all the *n* individuals (*n*=*n*_1_+*n*_2_+…+*n_h_*):

(2) $$\begin{array}{l} y=1\mu +X_{A}b_{A}+X_{D}b_{D}+X_{AA}b_{AA}+U_{E}e_{E}+\sum_{k=1}^{s}U_{A_{k}E}e_{A_{k}E}+\sum_{k=1}^{s}U_{D_{k}E}e_{D_{k}E}+\sum_{h=1}^{t}U_{{aa}_{h}E}e_{{AA}_{h}E}+e_{\varepsilon }\\ =\mathrm{Xb}+\sum_{u=1}^{r+1}U_{u}e_{u}\sim N(\mathrm{Xb},\;V=\sum_{u=1}^{r+1}\sigma_{u}^{2}U_{u}R_{u}U_{u}^{T}) \end{array}$$

where **y** is an *n*×1 vector of the observed phenotypic values, with *n* as the total number of individuals; **1** is an *n*×1 vector with all the elements=1; **b***_A_*=[*a*_1_ *a*_2_···*a_s_*]*^T^*, **b***_D_*=[*d*_1_ *d*_2_···*d_s_*]*^T^* and **b***_AA_*=[*aa*_1_ *aa*_2_···*aa_t_*]*^T^* are the parameter vectors for the fixed QTL effects with incidence matrixes **X***_A_*, **X***_D_* and **X***_AA_*, respectively; $e_{E}={\left[e_{1}e_{2}\cdots e_{p}\right]}^{T}\sim (0,\sigma_{E}^{2}I),e_{A_{k}E}={\left[{ae}_{k1}{ae}_{k2}\cdots {ae}_{kp}\right]}^{T}\sim (0,\sigma_{A_{k}E}^{2}R_{A_{k}E}),e_{D_{k}E}={\left[{de}_{k1}{de}_{k2}\cdots {de}_{kp}\right]}^{T}\sim (0,\sigma_{D_{k}E}^{2}R_{D_{k}E}),e_{{AA}_{h}E}={\left[{\mathrm{aae}}_{h1}\;{\mathrm{aae}}_{h2}\cdots {\mathrm{aae}}_{hp}\right]}^{T}\sim (0,\sigma_{{AA}_{h}E}^{2}R_{{AA}_{h}E})$ are random effect vectors with incidence matrixes **U***_E_*, **U**_*A*_*k*_*E*_, **U**_*D*_*k*_*E*_ and **U**_*AA*_*h*_*E*_, respectively; $e_{\varepsilon }\sim (0,\sigma_{\varepsilon }^{2}I)$ is the random vector of residual effects; and **R***_u_* is an identity matrix (*u*=1, 2, …, *r*+1) when there is no kinship between the original parents.

For any putative QTL, all the coefficients in model (2) are unknown; however, they can be substituted with their expectation inferred from the conditional probability of the QTL genotype given the two flanking markers. Let *p_ijk_* be the conditional probability for the QTL with *i* denoting the parental line (P*_i_*, *i*=1, 2), *j* denoting the genotype of flanking markers (*j*=1, 2, 3, 4), and *k* denoting the genotype of QTL (*k*=1, 2, 3). Conditional probabilities are calculated for the mixed population from two backcrosses between DH lines (or RI lines) and homozygous parents P_1_ and P_2_ (Table 1). Additionally, the coefficients of additive and dominance effects are 1 and −0.5 for genotype *Q_i_Q_i_*, 0 and 0.5 for *Q_i_q_i_*, −1 and −0.5 for *q_i_q_i_*, respectively. Thus the coefficients of the putative QTL effects in model (1) can be estimated by *x*_*A*_*k*__=(*p_ij_*_1_−*p_ij_*_3_) and *x*_*D*_*k*__=(*p_ij_*_2_−*p_ij_*_1_−*p_ij_*_3_)/2 for each progeny from the backcross between DH or RI lines and parent P*_i,_* and having the *j*th flanking marker genotype.

### 1.2 Strategy for detecting QTLs in the whole genome

Prior to fitting model (1), the numbers and locations of QTLs with genetic main effects or QE interaction effects should first be identified based on a marker linkage map and phenotypic values. After obtaining these effects, a systematic mapping strategy [22] for complex traits was used in the present study to estimate various parameters of QTLs.

- Mapping QTLs through 1D genome scan. According to the composite interval mapping (CIM) approach [23], mapping multiple QTLs can be simplified to 1D searching along a chromosome. It can be achieved by testing a QTL in a certain genomic region with some selected marker intervals as cofactors to control the background genetic effects from other QTLs outside of the region. Suppose *c* marker intervals (*M*_1−_*M*_1+_, *M*_2−_*M*_2+_,…, *M_c_*_−_*M_c_*_+_,) were selected as QTL candidate intervals, the model to test the QTL in locus *i* can be expressed as follows:
  (3) $$y_{hk}=\mu_{h}+a_{hi}x_{A_{ki}}+d_{hi}x_{D_{ki}}+\sum_{l}\left(\alpha_{hl}^{-}\xi_{kl}^{-}+\beta_{hl}^{-}\zeta_{kl}^{-}+\alpha_{hl}^{+}\xi_{kl}^{+}+\beta_{hl}^{+}\zeta_{kl}^{+}\right)+\varepsilon_{hk},$$
  where *μ_h_* is the population mean in the *h*th environment; *a_hi_* and *d_hi_* are the additive and dominance effects of *Q_i_* in the *h*th environment, respectively; $\alpha_{hl}^{-}\;(\alpha_{hl}^{+})$ and $\beta_{nl}^{-}\;(\beta_{nl}^{+})$ are the additive and the dominance effects of the *l*th marker interval in the *h*th environment, respectively; $\xi_{kl}^{-}\;(\xi_{kl}^{+})$ takes the value of 1, 0, −1 for genotype *M_l_M_l_*, *M_l_m_l_*, *m_l_m_l_* respectively, while $\zeta_{kl}^{-}\;(\zeta_{kl}^{+})$ takes the value of −0.5, 0.5, −0.5 for these three marker genotypes; the remaining variables and parameters have the same definitions as those in model (1).
- Mapping epistasis through 2D genome scan. After the 1D genome scan is completed with *t* main effect QTLs identified, a 2D genome scan is conducted to search for all possible epistasis including the effects of *t* QTLs and the interaction effects from *f* pre-selected paired marker intervals as the genetic background control in the model. The model to test the significance of epistatic interactions between loci *i* and *j* can be written as follows:
  (4) $$y_{hk}=\mu_{h}+{aa}_{\mathrm{hij}}x_{A_{ki}}x_{A_{kj}}+\sum_{s=1}^{t}\left(a_{hs}x_{A_{ks}}+d_{hs}x_{D_{ks}}\right)+\sum_{l}^{f}\left(\alpha \alpha_{hl}^{A^{-}B^{-}}\xi_{kl}^{A^{-}}\xi_{kl}^{B^{-}}+\alpha \alpha_{hl}^{A^{+}B^{+}}\xi_{kl}^{A^{+}}\xi_{kl}^{B^{+}}\right)+\varepsilon_{hk},$$
  where $\alpha \alpha_{hl}^{A^{-}B^{-}}(\alpha \alpha_{hl}^{A^{+}B^{+}})$ is the interaction effect between the flanking markers of interval *I^A^* and *I^B^*, (*I*^A−^, *I*^B−^) and (*I^A^*^+^, *I^B^*^+^). All other variables and parameters have the same definitions as those in equation (3).
- Hypothesis testing for significance of QTL effects.
  Both the genetic models of (3) and (4) can be reformatted into the following general multivariate linear mixed model in matrix form:
  (5) $$y=W_{\text{Q}}b_{\text{Q}}+W_{\text{B}}b_{\text{B}}+\varepsilon ,$$
  where, **b**_Q_ is a tested vector of QTL parameters (consisting of additive, dominance, and epistatic effects) with coefficient matrix **W**_Q_, **b**_B_ is the vector consisting of the population mean and the background effects due to the significant QTLs and marker intervals with the corresponding coefficient matrix **W**_B_. The *F*-statistic based on the Henderson III method [24] is used to test the significance of QTL effects, which can be expressed as follows
  $$F=\frac{\mathrm{SSR}(b_{\text{Q}}|b_{\text{B}})/(r_{W}-r_{W_{\text{B}}})}{\mathrm{SSE}/(n-r_{W})},$$
  where *r*_w_ is the rank of the matrix **W** = (**W**_Q_⋮**W**_B_) , and *r*_**W**_*B*__ is the rank of **W**_B_; *SSR*(**b**_Q_|**b**_B_) is the extra regression sum of squares attributed to the **b**_Q_ given the **b**_B_ in the genetic model; *SSE* is the residual sum of squares of the model (5). Under the null hypothesis *H*_0_:**b**_Q_=0, the *F*-statistic follows the *F* distribution with (*r*_W_→*r*_W_B__) and (*n*−*r*_w_)as the numerator and denominator degrees of freedom, respectively. For detailed information, refer to Yang et al. [22,25]

Prior to scanning the genome for candidate QTLs by models (3) and (4), the candidate marker interval or paired marker intervals with significant effects (additive, dominance, or epistatic effects) as cofactors in models (3) and (4) must first be selected via the method of marker pair selection (MPS) [26]. The same searching procedure proposed by Yang et al. [22] was used in this study.

### 1.3 Genome wide threshold value and the estimation of QTL genetic effects

We used the *F*-statistic based on the Henderson III method for a mixed linear model to determine the candidate marker intervals, as well as the QTLs with significant effects. The genome-wide threshold for the *F*-statistic was specified by the permutation procedure [27]. When the number of candidate QTLs and their positions in chromosome were obtained, the full model including effects of all candidate QTLs was constructed. The model selection procedure was adopted to further reduce the false positive rate of QTLs. Based on the result of model selection, the genetic effects of QTLs including QE interaction effects were estimated by the Bayesian method via Gibbs sampling [22].

## 2 Results

### 2.1 Simulation setting

A total of 200 Monte Carlo simulations for double back-cross populations with DH × P_1_ and DH × P_2_ progenies were conducted under three different environments to investigate the statistical properties of the proposed method in detecting QTL positions, estimating QTL effects, and predicting QE interactions. The size of mapping population was kept constant at 300 with three different ratios (1:1, 1:2, 1:5) of the number of DH × P_1_ population to the number of DH × P_2_ population, or equal ratios were used for the three populations with sizes of 180, 240, and 300. In all simulations, a genome of five chromosomes was constructed with 55 evenly distributed markers at a space of 10 cM. Suppose that 7 QTLs (denoted as Q1, Q2, Q3, Q4, Q5 Q6 and Q7), were involved in the genetic variation of a complex trait, of which 5 QTLs (Q1–Q5) had main genetic effects and 2 QTLs (Q6 and Q7) were pure epistatic QTLs. Five QTLs, Q1, Q3, Q5, Q6, and Q7, were involved in three pairs of epistatic effects denoted as EQ1 (Q1–Q6), EQ2 (Q3–Q5), and EQ3 (Q6–Q7), Only epistatic effects were set for EQ3. Q1 – Q5 were located on chromosomes 1–5, and Q6, Q7 on chromosomes 4 and 5, respectively. The heritability of a simulated trait was assumed to be 60%. Detailed configurations of parameters for each QTL are shown in Tables 2 and 3.

### 2.2 The potential bias in QTL parameter estimation arising from ignoring epistasis

A simulation comparison between two QTL models (with and without inclusion of epistatic effects) was performed to investigate the impact of epistases on estimating QTL main/epistatic effects, QE interactions, and QTL positions. Model I included both main and epistatic effects, while Model II did not include epistasis. Two populations with or without epistasis were simulated. Both populations had a sample size of 300 (150:150). The above-mentioned factors led to a total of four different cases: epistases existed and were included in the model (Case I), epistases existed but were ignored in the model (Case II), epistases did not exist and were included in the model (Case III), and epistases did not exist and were not included in the model (Case IV).

The results of the simulation clearly show that the mixed linear model approach provided largely unbiased estimates of QTL positions, QTL main/epistatic effects, and QE interactions (Tables 2 and 3), with small standard deviations. The results also revealed that the power of detecting an individual QTL ranged from 93% to 100%, showing the high efficiency of the proposed method.

The results under four different cases revealed that the positions of QTLs with main effects could be precisely estimated whether the genetic model included the epistases or not. In the presence of epistasis, cases I and II both provided unbiased parameter estimates with minor differences for additive effects; similarly, when there were no QTL epistases, almost the same results were observed for the cases III and IV regardless of whether epistases were included in the model or not (Table 2). However, in the presence of epistasis, Case I tended to give much better estimates of dominance and interaction effects with the environment, compared with Case II (Table 2).

For estimating epistases, it should be noted that epistases can only be identified when they exist and when an epistatic model is used (Case I). In the other cases (Case II, Case III and Case IV), either using a model that omits epistasis (Case II and Case IV) or the absence of epistases (Case III and Case IV), will both result in undetectable epistatic effects. However, the results in Table 3 revealed that epistatic parameters were well estimated by the epistatic model (Case I), although the statistical power was relatively lower than that of individual QTLs. Considering that the estimation accuracy of QTL parameters is affected by epistases, and that epistases are generally involved in genetic variation of quantitative trait, a model that includes epistasis is preferable for use in QTL studies.

### 2.3 Impact of heritability and population constitution on estimation of QTL parameters

To investigate the impacts of heritability and population size on estimating QTL parameters, we conducted simulations with the several scenarios described in Tables 4 and 5. Model I with epistases (see Section 2.2) was used for three sample populations with different sizes and a constitution ratio of 1:1, that is; 180 (90:90), 240 (120:120), and 300 (150:150). The simulation results revealed that including epistases in the model was essential to reliably detect and quantify QTLs with the mixed linear model approach. The large heritability and population size could increase the statistical power and estimation precision of the QTL parameters. As shown in Table 4, the statistical power of every QTL with main genetic effects was nearly 100%, except for Q5 with relatively small heritability; this finding indicated that heritability is an important factor in QTL detection. In addition, as the population size increased to 240, most QTLs with main effects could be distinguished efficiently. For the epistatic interaction (Table 5), the statistical power and accuracy of epistatic parameters were improved with higher heritability or larger population sizes. Under all of the scenarios, had fewer type II errors than other two paired epistatic QTLs. Meanwhile, for each pair of epistatic QTLs, increased population size resulted in greater predictive power and more accurate estimates of parameters.

In addition to heritability and sample size, the population constitution may be another important factor affecting QTL mapping. Thus, using the same model as above (Model I), we performed another series of Monte Carlo simulations for mapping populations with the same size but three different constitutions, 300 (150:150), 300 (100:200), and 300 (50:250). As shown in Tables 6 and 7, the population with even proportions improved the accuracy of estimating the genetic effects of QTLs. In most cases, the estimates of parameters were slightly more accurate in the evenly distributed sample, as indicated by standard deviation, although the differences were not so clear (Tables 6 and 7). The false discovery rate also tended to increase with a more unevenly distributed population (0.0416, 0.0537 to the 0.0543 for three respective population constitutions; Table 7). In all cases, the power for the main QTL was higher than that for epistasis. Thus, a double backcross population with a large sample size and evenly distributed population is preferred and will enhance the accuracy of QTL analysis.

## 3 Discussion

Backcrossing is a very commonly used breeding method. It is widely used in plant breeding to precisely improve the target trait by transferring the associated gene(s) from the donor parent to the recurrent parent, to control hybrid populations, and to overcome the barriers of distant crosses, and so on. Backcrossing is also a very useful design for dissecting the genetic mechanism of quantitative traits, and has been used in mapping QTLs for complex traits in crops and animals [28,29]. Advanced backcrossing has been used for fine mapping of QTLs [30,31]. However, when a traditional temporary BC population derived from crossing F_1_ with one of its parents is used to map QTLs for complex traits, it is impossible to obtain different phenotypes of each genotype in different environments. In the present study, we proposed mapping of QTLs based on an immortal double backcross population (that is, a population constructed by mating DH or RI lines with homozygous parents P1 and P2 separately). Because of the benefits of the identical genetic constitution of the mapping population and repeated multiple environment experiments, QTL and QE interactions can be analyzed accurately. Meanwhile, time and labor costs can be saved because the molecular genotype of each individual can be inferred from the molecular information of the parents and the DH or RI lines. Another advantage is that the double backcross population may improve the statistical power and precision of estimation, such as estimates of QTL positions, additive effects, and dominance effects, because of the increased population size and more abundant molecular genotypes at one locus or multiple loci. In QTL mapping, an F_2_ population is ideal for analyzing additive, dominance, and additive-additive epistasis of QTLs because it has richer genetic diversity than DH, BC, and RI populations. On the other hand, to overcome the shortcomings resulting from heterozygosity of individuals and the need to conduct repeated experiments, the permanent or immortal F_2_ (PF2 or IF2) populations derived from mating of DH or RI lines is proposed to mimic the F_2_ population. The double backcross population is also an excellent mimic of the F_2_ population, although it has fewer genotype types of multiple loci than the F_2_ population, and of the four types of epistases, only additive-additive epistasis can be analyzed. However, there are still many merits of the double backcross mapping population for QTL studies in crops and especially in animals.

Recent studies of QTL mapping in rice and maize have shown that dominance and epistasis, especially the additive × additive interaction, play a key role in contributing to heterosis [10,32,33]. In the conventional BC population, the additive and dominant effects can only be identified in a mixture, and the additive × additive interaction cannot be detected separately. The double backcross population proposed in the present study can be used to tackle this problem, and accurately and efficiently estimates additive, dominance, and additive × additive interaction effects. However, population structure should be taken into account when mapping QTLs in the double backcross population. A population with equal ratios of the two backcross populations has a more similar genetic structure to the F_2_ population, and should show greater statistical power and estimation accuracy compared with those of other populations. However, our simulation did not show large differences among three populations consisting of 150 and 150 lines, 100 and 200 lines, and 50 and 250 lines derived from crosses of DH × P_1_ and DH × P_2_. When there are extreme changes in proportions of the population, the double-backcross populations will become more similar to a traditional one-backcross population, and the coefficient matrix of the model will become singular; thus, some genetic effects of QTLs, such as dominance, cannot be analyzed. Thus, an evenly distributed population is preferable and may improve the precision of estimates and statistical power.

It is believed that epistatic interactions are important genetic buffers that provide functional redundancy for species to survive under adverse changes in their environment. As well, epistatic interactions could generate more phenotypic polymorphisms in response to natural and artificial selection [34]. So far, there is much research evidence on the role of epistatic interactions. However, to detect a pair of true positive epistatic QTLs usually requires a relatively large population. Actually, the size of the simulation population, 300, was large enough to detect epistasis under the simulation scenarios considered here. As shown in Table 7, irrespective of various population proportions, the false positive rates of epistasis detection are quite low, 0.04, 0.05, and 0.05 in the three surveyed cases. On the other hand, the simulation study suggests that if epistasis of the QTLs does contribute to a trait, it will affect the accuracy of estimated QTL parameters. When there is significant epistasis in agronomic traits, mapping QTLs with case II will lead to inferior estimates of the effects and positions. Therefore, the best strategy is to include epistatic effects in a QTL model, especially when some epistases are actually involved in genetic variation of complex traits.

## Figures and Tables

**Table 1:** Conditional probabilities of QTL for two backcrosses populations from mating between DH (or RI) lines and parental lines (P_1_ and P_2_)

Backcross Types	Marker genotypes	Expected frequencya)					
		DH			RI		
		*Q_i_Q_i_*	*Q_i_q_i_*	*q_i_q_i_*	*Q_i_Q_i_*	*Q_i_q_i_*	*q_i_q_i_*
DH or RI×P_1_	*M_i_*_−_*M_i_*_−_*M_i+_M_i+_*	*s*_1_*s*_2_/*s*	*r*_1_*r*_2_/*s*	0	$\frac{1}{1+4r_{1}r_{2}}$	$\frac{4r_{1}r_{2}}{1+4r_{1}r_{2}}$	0
	*M_i_*_−_*M_i_*_−_*M_i+_M_i+_*	*s*_1_*r*_2_/*r*	*r*_1_*s*_2_/*r*	0	*r*_2_/(*r*_1_+*r*_2_)	*r*_1_/(*r*_1_+*r*_2_)	0
	*M_i_*_−_*m_i_*_−_*M_i+_M_i+_*	*r*_1_*s*_2_/*r*	*s*_1_*r*_2_/*r*	0	*r*_1_/(*r*_1_+*r*_2_)	*r*_2_/(*r*_1_+*r*_2_)	0
	*M_i_*_−_*m_i_*_−_*M_i+_m_i+_*	*r*_1_*r*_2_/*s*	*s*_1_*s*_2_/*s*	0	$\frac{4r_{1}r_{2}}{1+4r_{1}r_{2}}$	$\frac{1}{1+4r_{1}r_{2}}$	0
DH or RI×P_2_	*M_i_*_−_*m_i_*_−_*M_i+_m_i+_*	0	*s*_1_*s*_2_/*s*	*r*_1_*r*_2_/*s*	0	$\frac{1}{1+4r_{1}r_{2}}$	$\frac{4r_{1}r_{2}}{1+4r_{1}r_{2}}$
	*M_i_*_−_*m_i_*_−_*m_i+_m_i+_*	0	*s*_1_*r*_2_/*r*	*r*_1_*s*_2_/*r*	0	*r*_2_/(*r*_1_+*r*_2_)	*r*_1_/(*r*_1_+*r*_2_)
	*m_i_*_−_*m_i_*_−_*M_i+_m_i+_*	0	*r*_1_*s*_2_/*r*	*s*_1_*r*_2_/*r*	0	*r*_1_/(*r*_1_+*r*_2_)	*r*_2_/(*r*_1_+*r*_2_)
	*m_i_*_−_*m_i_*_−_*m_i+_m_i+_*	0	*r*_1_*r*_2_/*s*	*s*_1_*s*_2_/*s*	0	$\frac{4r_{1}r_{2}}{1+4r_{1}r_{2}}$	$\frac{1}{1+4r_{1}r_{2}}$

a The putative **QTL** (*Q_i_*) and two flanking marker are ordered along the chromosome as *M_i_*_−_→*Q_i_*→*M_i+_*_,_ where the recombination rate between *M_i_*_−_ and *M_i+_* is denoted as *r*, the one between *M_i_*_−_ and *Q_i_* denoted as *r*_1_, and the one between *Q_i_* and *M_i+_* denoted as *r*_2_; *s*=1−*r*, *s*_1_=1−*r*_1_, and *s*_2_=1−*r*_2_.

**Table 2:** Comparisons of the estimation of QTL parameter and power by models with and without epistasesa)

QTL	Case	Position	*A*	*D*	*AE1*	*AE2*	*AE3*	*DE1*	*DE2*	*DE3*	Power
Q1	Expected	44.0	−2.78	−3.08	0.00	0.00	0.00	0.00	0.00	0.00	
	I	43.74(2.86)	−2.67(0.31)	−2.9(0.52)	−0.00(0.78)	−0.04(0.81)	0.05(0.84)	−0.07(0.31)	0.00(0.18)	0.07(0.32)	98.0
	II	43.72(2.87)	−2.69(0.33)	−1.49(1.62)	−0.01(0.79)	−0.04(0.80)	0.03(0.85)	−1.04(1.12)	0.07(0.27)	0.97(1.03)	98.0
	III	44.02(2.13)	−2.69(0.35)	−2.99(0.35)	−0.00(0.84)	−0.06(0.85)	0.04(0.87)	−0.01(0.16)	0.00(0.16)	0.01(0.17)	100
	IV	44.02(2.13)	−2.69(0.33)	−2.99(0.35)	−0.00(0.84)	−0.06(0.85)	0.04(0.87)	−0.00(0.16)	−0.00(0.14)	0.01(0.15)	100
Q2	Expected	75.0	0.00	2.50	−2.00	−0.70	2.70	0.00	0.00	0.00	
	I	75.36(2.75)	0.00(0.31)	2.36(0.32)	−1.90(0.98)	−0.65(1.01)	2.54(1.03)	−0.01(0.16)	0.00(0.13)	0.00(0.15)	99.5
	II	75.33(2.75)	−0.03(0.33)	2.37(0.35)	−1.86(0.97)	−0.60(1.00)	2.58(1.05)	−0.01(1.16)	0.00(0.12)	0.01(0.16)	99.5
	III	75.33(2.48)	0.00(0.26)	2.34(0.35)	−1.89(0.99)	−0.64(0.98)	2.55(1.05)	0.00(0.14)	0.00(0.13)	0.00(0.14)	100
	IV	75.33(2.48)	0.00(0.25)	2.34(0.34)	−1.88(0.99)	−0.63(0.98)	2.55(1.06)	0.00(0.15)	0.00(0.14)	−0.00(0.14)	100
Q3	Expected	50.0	2.90	3.50	2.60	−1.30	−1.30	0.00	0.00	0.00	
	I	49.91(0.69)	2.53(0.46)	2.92(0.83)	2.56(0.88)	−1.30(0.90)	−1.18(0.86)	0.26(0.52)	−0.01(0.23)	−0.24(0.51)	99.5
	II	49.91(0.69)	2.57(0.44)	2.12(1.43)	2.51(0.88)	−1.31(0.91)	−1.18(0.88)	0.88(0.96)	−0.05(0.27)	−0.84(0.91)	99.5
	III	49.92(0.62)	2.53(0.43)	3.18(0.44)	2.57(0.88)	−1.31(0.89)	−1.18(0.86)	−0.00(0.14)	0.01(0.15)	−0.01(0.13)	100
	IV	49.92(0.62)	2.53(0.43)	3.17(0.43)	2.57(0.88)	−1.31(0.89)	−1.18(0.86)	−0.01(0.14)	0.01(0.15)	−0.00(0.12)	100
Q4	Expected	73.0	−3.30	−1.90	0.00	0.00	0.00	−2.10	2.50	−0.40	
	I	74.20(2.85)	−3.12(0.49)	−1.73(0.41)	0.00(0.63)	−0.03(0.69)	0.02(0.69)	−1.91(0.44)	2.29(0.50)	−0.37(0.34)	100
	II	74.21(2.85)	−3.12(0.50)	−1.74(0.42)	0.00(0.62)	−0.03(0.68)	0.02(0.68)	−1.90(0.45)	2.29(0.51)	−0.35(0.83)	100
	III	73.59(2.20)	−3.18(0.40)	−1.79(0.32)	0.00(0.81)	−0.05(0.87)	0.05(0.86)	−1.93(0.37)	2.35(0.41)	−0.42(0.29)	100
	IV	73.59(2.20)	−3.18(0.40)	−1.79(0.31)	0.00(0.81)	−0.05(0.87)	0.05(0.86)	−1.93(0.37)	2.35(0.40)	−0.42(0.29)	100
Q5	Expected	15.0	1.90	0.00	0.00	0.00	0.00	0.00	0.00	0.00	
	I	14.68(3.78)	1.82(0.28)	−0.17(0.58)	0.00(0.75)	0.01(0.76)	0.01(0.77)	0.19(0.44)	−0.03(0.22)	−0.15(0.41)	93.0
	II	14.72(3.79)	1.84(0.31)	−0.96(1.03)	−0.02(0.73)	0.01(0.76)	0.01(0.76)	0.83(0.91)	−0.08(0.28)	−0.73(0.83)	93.0
	III	15.10(3.94)	1.82(0.25)	0.02(0.27)	−0.01(0.81)	−0.00(0.84)	0.02(0.86)	0.01(0.15)	−0.02(0.16)	0.01(0.16)	92.5
	IV	15.10(3.94)	1.82(0.25)	0.02(0.26)	−0.01(0.81)	−0.00(0.84)	0.01(0.86)	0.00(0.15)	−0.02(0.14)	0.01(0.14)	92.5

a) Q1, Q2, Q3, Q4, and Q5 denote five QTLs with main effects; I, II, III, IV denote QTL models with or without inclusion of epistases when epistatic effects exist or do not exist, respectively; Expected stand for the true value of parameter; *A*, *D*, *AE*, *DE* are additive, dominance and their interaction effects with environment, numbers in parenthesis are their corresponding standard deviation of estimates. Power is probability of identifying QTLs in 200 simulations. Size of mapping population was 300, consisting of 150 lines of DH×P_1_ and 150 lines of DH×P_2_.

**Table 3:** Estimate of parameter for paired epistatic QTLs and power by the model with epistasesa)

Epistatic QTLs	QTL_*i*	QTL_*j*	*AA*	*AAE1*	*AAE2*	*AAE3*	Power
EQ1(Q1–Q6)	44.0b)	24.0	−3.09	2.52	−0.16	−2.36	
	43.75(2.85)c)	23.08(2.96)	−2.79(0.65)	2.17(0.70)	−0.15(0.40)	−2.03(0.68)	97.0
EQ2(Q3–Q5)	50.0	15.0	2.10	−2.10	0.20	1.90	
	49.90(0.72)	14.79(3.68)	2.05(0.55)	−1.87(0.52)	0.12(0.39)	1.72(0.51)	74.5
EQ3(Q6–Q7)	24.0	79.0	−2.60	0.00	0.00	0.00	
	23.10(2.89)	79.37(3.22)	−2.54(0.45)	−0.01(0.24)	−0.04(0.19)	0.05(0.28)	55.0

a) Three pairs of epistatic interaction, EQ1, EQ2, and EQ3, consist of Q1 and Q6, Q3 and Q5, and Q6 and Q7, respectively;

b) true value of parameter;

c) estimate of parameter and standard deviation (in parenthesis). *AA, AAE* are additive-additive epistases and their interaction effects with environment. Power is probability of identifying QTLs in 200 simulations. Size of mapping population is 300, consisting of 150 lines of DH×P_1_ and 150 lines of DH×P_2_.

**Table 4:** Estimation of QTL effects and their interaction effects with environments under three different sample sizes

QTL (heritability %)	Population	Position	*A*	*D*	*AE1*	*AE2*	*AE3*	*DE1*	*DE2*	*DE3*	Power
Q1 (11.03)	Expected	44.0a)	−2.78	−3.08	0.00	0.00	0.00	0.00	0.00	0.00	
	I	44.15(3.68)b)	−2.63(0.46)	−2.49(1.09)	−0.07(0.88)	0.10(0.93)	−0.02(0.93)	−0.33(0.67)	0.04(0.31)	0.30(0.64)	94
	II	44.21(3.78)	−2.66(0.37)	−2.74(0.70)	0.00(0.81)	0.04(0.78)	−0.03(0.83)	−0.12(0.38)	−0.01(0.20)	0.13(0.37)	99
	III	43.83(3.06)	−2.64(0.36)	−2.88(0.55)	0.01(0.79)	−0.05(0.82)	0.05(0.84)	−0.06(0.27)	−0.00(0.18)	0.06(0.29)	97.5
Q2 (7.68)	Expected	75.0	0.00	2.45	−2.00	−0.70	2.70	0.00	0.00	0.00	
	I	75.17(3.44)	0.00(0.41)	2.41(0.44)	−1.93(1.08)	−0.51(1.06)	2.48(1.21)	−0.03(0.18)	0.01(0.14)	0.02(0.18)	99
	II	75.16(2.95)	0.01(0.34)	2.41(0.33)	−1.88(0.99)	−0.67(0.87)	2.58(1.09)	−0.01(0.18)	−0.00(0.16)	0.02(0.20)	99
	III	75.61(2.77)	0.00(0.32)	2.36(0.33)	−1.89(1.00)	−0.66(1.03)	2.54(1.04)	−0.01(0.16)	0.00(0.14)	0.01(0.15)	99
Q3 (16.53)	Expected	50.0	2.90	3.52	2.60	−1.30	−1.30	0.00	0.00	0.00	
	I	49.92(1.48)	2.90(0.41)	2.89(0.96)	2.42(1.01)	−1.14(0.98)	−1.27(0.98)	0.54(0.77)	−0.05(0.30)	−0.48(0.70)	99.5
	II	50.13(1.41)	2.52(0.52)	2.82(0.94)	2.51(0.94)	−1.30(0.91)	−1.16(0.90)	0.35(0.62)	−0.05(0.25)	−0.30(0.53)	100
	III	49.95(0.65)	2.90(0.28)	3.26(0.64)	2.55(0.91)	−1.32(0.92)	−1.16(0.89)	0.28(0.54)	−0.02(0.23)	−0.26(0.52)	99.5
Q4 (12.65)	Expected	73.0	−3.31	−1.87	0.00	0.00	0.00	−2.10	2.50	−0.40	
	I	73.82(3.50)	−3.10(0.53)	−1.72(0.49)	−0.05(0.68)	0.11(0.73)	−0.07(0.70)	−1.93(0.55)	2.19(0.58)	−0.24(0.52)	96
	II	73.92(2.86)	−3.19(0.37)	−1.80(0.37)	−0.02(0.67)	−0.03(0.63)	0.04(0.69)	−1.94(0.38)	2.28(0.44)	−0.30(0.38)	100
	III	74.18(2.97)	−3.10(0.51)	−1.72(0.41)	−0.00(0.64)	−0.03(0.71)	0.02(0.69)	−1.89(0.46)	2.28(0.51)	−0.36(0.34)	100
Q5 (2.36)	Expected	15.0	1.92	0.00	0.00	0.00	0.00	0.00	0.00	0.00	
	I	14.86(5.15)	1.88(0.35)	−0.43(0.83)	−0.11(0.79)	0.08(0.80)	0.04(0.79)	0.53(0.81)	−0.06(0.30)	−0.47(0.71)	64
	II	15.38(3.99)	1.82(0.33)	−0.23(0.70)	0.02(0.77)	−0.07(0.78)	0.06(0.77)	0.24(0.49)	−0.01(0.25)	−0.23(0.46)	92.5
	III	14.54(3.90)	1.82(0.29)	−0.16(0.61)	−0.01(0.76)	0.02(0.78)	0.01(0.79)	0.20(0.45)	−0.03(0.23)	−0.17(0.43)	90.5

a) True values of QTL parameter;

b) estimates of QTL parameter and their standard deviation in parenthesis; *A, D, AE, DE* denote estimates of QTL additive, dominance effects and their interaction effects with environments, respectively; I, II and III represent three different population sizes; 180 (90 from DH×P_1_ and 90 from DH×P_2_), 240 (120 from DH×P_1_ and 120 from DH×P_2_) and 300 (150 from DH×P_1_ and 150 from DH×P_2_), respectively.

**Table 5:** Estimation of epistatic effects and their interaction effects with environments under three different sample sizes

Epistatic QTL (heritability %)	Population	Position_*i*	Position_*j*	*AA*	*AAE1*	*AAE2*	*AAE3*	Power
EQ1 (4.98)	Expected	44.0a)	24.0	−3.09	2.52	−0.16	−2.36	
	I	44.27(3.45)b)	22.30(4.13)	−2.93(0.74)	2.08(0.78)	−0.21(0.59)	−1.87(0.82)	64.5
	II	44.50(3.39)	22.99(3.18)	−2.78(0.70)	2.15(0.76)	−0.19(0.41)	−1.95(0.71)	90.5
	III	43.82(2.96)b)	23.07(3.03)	−2.81(0.67)	2.15(0.73)	−0.13(0.43)	−2.02(0.69)	96.0
EQ2 (2.67)	Expected	50.0	15.0	2.10	−2.10	0.20	1.90	
	I	50.22(1.07)	14.41(3.83)	2.33(0.76)	−1.73(0.90)	0.13(0.55)	1.57(0.86)	27
	II	50.10(1.19)	15.55(3.77)	2.16(0.54)	−1.96(0.54)	0.23(0.41)	1.69(0.56)	63
	III	49.96(0.58)	14.48(3.96)	2.07(0.58)	−1.85(0.57)	0.13(0.38)	1.69(0.55)	72.5
EQ3 (2.10)	Expected	24.0	79.0	−2.60	0.00	0.00	0.00	
	I	23.09(4.21)	79.94(3.32)	−2.72(0.51)	0.05(0.25)	0.05(0.20)	−0.10(0.22)	17.5
	II	23.21(2.81)	79.34(3.43)	−2.60(0.42)	0.03(0.29)	−0.02(0.27)	−0.01(0.23)	38
	III	23.15(2.87)	79.44(3.33)	−2.54(0.46)	−0.02(0.23)	−0.06(0.20)	0.07(0.27)	50.5

a) True values of QTL parameter;

b) estimates of QTL parameter and standard deviation in parenthesis; *AA* and *AAE* denote estimates of additive by additive effects and their interaction effects with environments, respectively; I, II and III have same definition as that in Table 4, with the false discovery rates of 0.103, 0.049, and 0.050, respectively.

**Table 6:** Estimation of QTL effects under three different constitutions of mapping population

QTL (heritability %)	Population	Position	*A*	*D*	*AE1*	*AE2*	*AE3*	*DE1*	*DE2*	*DE3*	Power
Q1 (11.03)	Expected	a)44.0	−2.78	−3.08	0.00	0.00	0.00	0.00	0.00	0.00	
	I	b)43.83(3.06)	−2.64(0.36)	−2.88(0.55)	0.01(0.79)	−0.05(0.82)	0.05(0.84)	−0.06(0.27)	−0.00(0.18)	0.06(0.29)	97.5
	II	44.05(2.75)	−2.63(0.43)	−2.79(0.65)	0.04(0.90)	−0.06(0.89)	0.02(0.93)	−0.14(0.45)	0.03(0.30)	0.10(0.45)	100
	III	44.13(2.18)	−2.64(0.53)	−2.91(0.67)	−0.09(0.90)	−0.02(0.85)	0.10(0.91)	−0.11(0.64)	0.03(0.54)	0.08(0.60)	100
Q2 (7.68)	Expected	75.0	0.00	2.45	−2.00	−0.70	2.70	0.00	0.00	0.00	
	I	75.61(2.77)	0.00(0.32)	2.36(0.33)	−1.89(1.00)	−0.66(1.03)	2.54(1.04)	−0.01(0.16)	0.00(0.14)	0.01(0.15)	99
	II	75.33(2.98)	0.02(0.32)	2.36(0.39)	−1.79(1.04)	−0.67(1.00)	2.49(1.08)	−0.05(0.29)	0.01(0.31)	0.03(0.30)	99
	III	75.14(2.71)	−0.03(0.45)	2.34(0.49)	−1.95(1.06)	−0.63(1.02)	2.60(1.17)	−0.05(0.65)	0.02(0.69)	0.03(0.68)	100
Q3 (16.53)	Expected	50.0	2.90	3.52	2.60	−1.30	−1.30	0.00	0.00	0.00	
	I	49.95(0.65)	2.90(0.28)	3.26(0.64)	2.55(0.91)	−1.32(0.92)	−1.16(0.89)	0.28(0.54)	−0.02(0.23)	−0.26(0.52)	99.5
	II	49.98(0.83)	2.93(0.29)	3.04(0.80)	2.65(0.97)	−1.32(0.89)	−1.28(0.92)	0.44(0.76)	−0.04(0.33)	−0.40(0.69)	100
	III	50.00(0.77)	2.94(0.37)	2.89(0.91)	2.53(1.07)	−1.25(0.98)	−1.24(1.06)	0.68(1.05)	−0.08(0.66)	−0.59(1.00)	100
Q4 (12.65)	Expected	73.0	−3.31	−1.87	0.00	0.00	0.00	−2.10	2.50	−0.40	
	I	74.18(2.97)	−3.10(0.51)	−1.72(0.41)	−0.00(0.64)	−0.03(0.71)	0.02(0.69)	−1.89(0.46)	2.28(0.51)	−0.36(0.34)	100
	II	74.12(2.52)	−3.15(0.48)	−1.80(0.39)	0.02(0.75)	0.00(0.73)	−0.04(0.72)	−1.94(0.52)	2.30(0.56)	−0.35(0.42)	100
	III	74.38(2.12)	−3.21(0.46)	−1.84(0.49)	−0.15(0.77)	0.05(0.82)	0.09(0.78)	−1.87(0.69)	2.29(0.72)	−0.39(0.64)	100
Q5 (2.36)	Expected	15.0	1.92	0.00	0.00	0.00	0.00	0.00	0.00	0.00	
	I	14.54(3.90)	1.82(0.29)	−0.16(0.61)	−0.01(0.76)	0.02(0.78)	0.01(0.79)	0.20(0.45)	−0.03(0.23)	−0.17(0.43)	90.5
	II	14.21(4.14)	1.86(0.29)	−0.24(0.60)	0.09(0.81)	0.04(0.77)	−0.12(0.79)	0.21(0.49)	−0.04(0.27)	−0.17(0.48)	74.5
	III	15.52(4.89)	1.93(0.40)	−0.06(0.62)	−0.11(0.78)	0.03(0.97)	0.09(0.87)	0.32(0.78)	0.01(0.72)	−0.33(0.79)	42.0

a) True values of QTL parameter;

b) estimates of QTL parameter and standard deviation in parenthesis; *A, D, AE, DE* denote the estimates of QTL additive, dominance effects and their interaction effects with environments, respectively; I,II and III represent three different constitutions of population, 300 (150 from DH×P_1_ and 150 from DH×P_2_), 300 (100 from DH×P_1_ and 200 from DH×P_2_) and 300 (50 DH×P_1_ and 250 from DH×P_2_), respectively.

**Table 7:** Estimation of epistatic effects and their interaction effects with environments under three different constitutions of mapping population

Epistatic QTL (heritability %)	Population	Position_*i*	Positiona_*j*	*AA*	*AAE1*	*AAE2*	*AAE3*	Power
EQ1 (4.98)	Expected	44.0a)	24.0	−3.09	2.52	−0.16	−2.36	
	I	43.82(2.96)b)	23.07(3.03)	−2.81(0.67)	2.15(0.73)	−0.13(0.43)	−2.02(0.69)	96.0
	II	44.09(2.60)	22.89(3.35)	−2.78(0.73)	2.11(0.75)	−0.14(0.47)	−1.96(0.72)	96.0
	III	44.04(2.07)	23.23(3.19)	−2.79(0.70)	2.03(0.87)	−0.13(0.51)	−1.91(0.83)	99.0
EQ2 (2.67)	Expected	50.0	15.0	2.10	−2.10	0.20	1.90	
	I	49.96(0.58)	14.48(3.96)	2.07(0.58)	−1.85(0.57)	0.13(0.38)	1.69(0.55)	72.5
	II	49.93(0.97)	14.25(4.20)	1.98(0.48)	−1.85(0.64)	0.21(0.44)	1.61(0.57)	56.5
	III	49.94(0.82)	15.21(4.86)	2.07(0.61)	−1.96(0.73)	0.25(0.58)	1.69(0.65)	34.0
EQ3 (2.10)	Expected	24.0	79.0	−2.60	0.00	0.00	0.00	
	I	23.15(2.87)	79.44(3.33)	−2.54(0.46)	−0.02(0.23)	−0.06(0.20)	0.07(0.27)	50.5
	II	23.27(3.16)	80.22(3.53)	−2.50(0.41)	0.06(0.31)	−0.03(0.28)	−0.04(0.21)	50.0
	III	23.33(3.19)	79.62(3.30)	−2.43(0.41)	−0.01(0.22)	−0.02(0.20)	0.03(0.24)	58.5

a) True values of the positions, additive-additive epistatic effects and their interaction with environment for paired epistatic QTLs;

b) estimate of parameter and standard deviation in parenthesis; *AA* and *AAE* denote estimates of additive by additive effects and their interaction effects with environments, respectively; I,II and III have same definition as that in Table 6, with false discovery rates of 0.0416, 0.0537, 0.0543, respectively.
